# Tropical Meridional Overturning Circulation Observed by Subsurface Moorings in the Western Pacific

**DOI:** 10.1038/s41598-018-26047-7

**Published:** 2018-05-16

**Authors:** Lina Song, Yuanlong Li, Jianing Wang, Fan Wang, Shijian Hu, Chuanyu Liu, Xinyuan Diao, Cong Guan

**Affiliations:** 1 CAS Key Laboratory of Ocean Circulation and Waves, Institute of Oceanology, Chinese Academy of Sciences, Qingdao, China; 2 Institute of Oceanographic Instrumentation, Shandong Academy of Sciences, Qingdao, China; 3 Function Laboratory for Ocean Dynamics and Climate, Qingdao National Laboratory for Marine Science and Technology, Qingdao, China

## Abstract

Meridional ocean current in the northwestern Pacific was documented by seven subsurface moorings deployed at 142°E during August 2014-October 2015. A sandwich structure of the tropical meridional overturning circulation (TMOC) was revealed between 0–6°N that consists of a surface northward flow (0–80 m), a thermocline southward flow (80–260 m; 22.6–26.5 *σ*_*θ*_), and a subthermocline northward flow (260–500 m; 26.5–26.9 *σ*_*θ*_). Based on mooring data, along with satellite and reanalysis data, prominent seasonal-to-interannual variations were observed in all three layers, and the equatorial zonal winds were found to be a dominant cause of the variations. The TMOC is generally stronger in boreal winter and weaker in summer. During 2014–2015, the TMOC was greatly weakened by westerly wind anomalies associated with the El Niño condition. Further analysis suggests that the TMOC can affect equatorial surface temperature in the western Pacific through anomalous upwelling/downwelling and likely plays a vital role in the El Niño-Southern Oscillation (ENSO).

## Introduction

The shallow meridional overturning circulation (MOC) is a primary component of the Pacific wind-driven circulation^1^. Embedded within the subtropical cells that connect the tropical and subtropical ocean, the tropical MOC (TMOC) exists within the tropical ocean and consists of upwelling at the equator, a surface poleward flow, off-equatorial downwelling at ~5°, and an equatorial return flow in the thermocline^2,3^. The TMOC acts as an important agent for heat exchange between the equatorial and off-equatorial ocean^4^ and thereby plays a significant role in the recharge–discharge regime of the El Niño–Southern Oscillation (ENSO)^5^. It is primarily driven by basin-wide easterly trade winds near the equator, forming a closed cell pair off the Pacific equator^6–8^. The thermocline cold water brought by the lower branch of the TMOC feeds the eastward-flowing Equatorial Undercurrent (EUC) and affects the tropical Pacific sea surface temperature (SST) through equatorial upwelling^9–11^. Therefore, variability in either the strength or temperature of the TMOC may exert a significant impact on the tropical Pacific climate^12,13^.

However, neither the climatological structure nor the temporal variability of the Pacific TMOC is well documented due to the shortage of direct observations. The complex flow structure of the TMOC covers only a few degrees of latitude and therefore presents a challenge for *in situ* observation. As a result, existing knowledges of the TMOC were mainly established by synoptic cruise measurements and model simulations^14–18^. It is of paramount need to verify and complement these knowledges by long-term, *in situ* observations with sufficient spatial and temporal resolutions.

In particular, most previous studies focused on the TMOC in the central-to-eastern Pacific basin, and very few observations are available for the western Pacific counterpart. Equatorial upwelling of the western Pacific TMOC may contribute to SST variability of the warm pool and atmospheric deep convection^19^. It remains unknown whether the western Pacific TMOC shows behaviors similar to those of the basin-mean TMOC or whether its spatial-temporal characteristics are unique. Moreover, the effect of TMOC variability on regional SST has not yet been examined. In this study, we aim to describe and understand the structure and variability of the northwest Pacific component of the TMOC and its impact on local SST. Acoustic Doppler current profiler (ADCP) measurements collected from seven subsurface moorings at 142°E during August 2014-October 2015 provided continuous records of meridional current in the upper kilometer of the ocean, allowing a thorough investigation of the TMOC with unprecedented data quality and coverage.

## Results

### Structure of the TMOC

**Figure 1:**
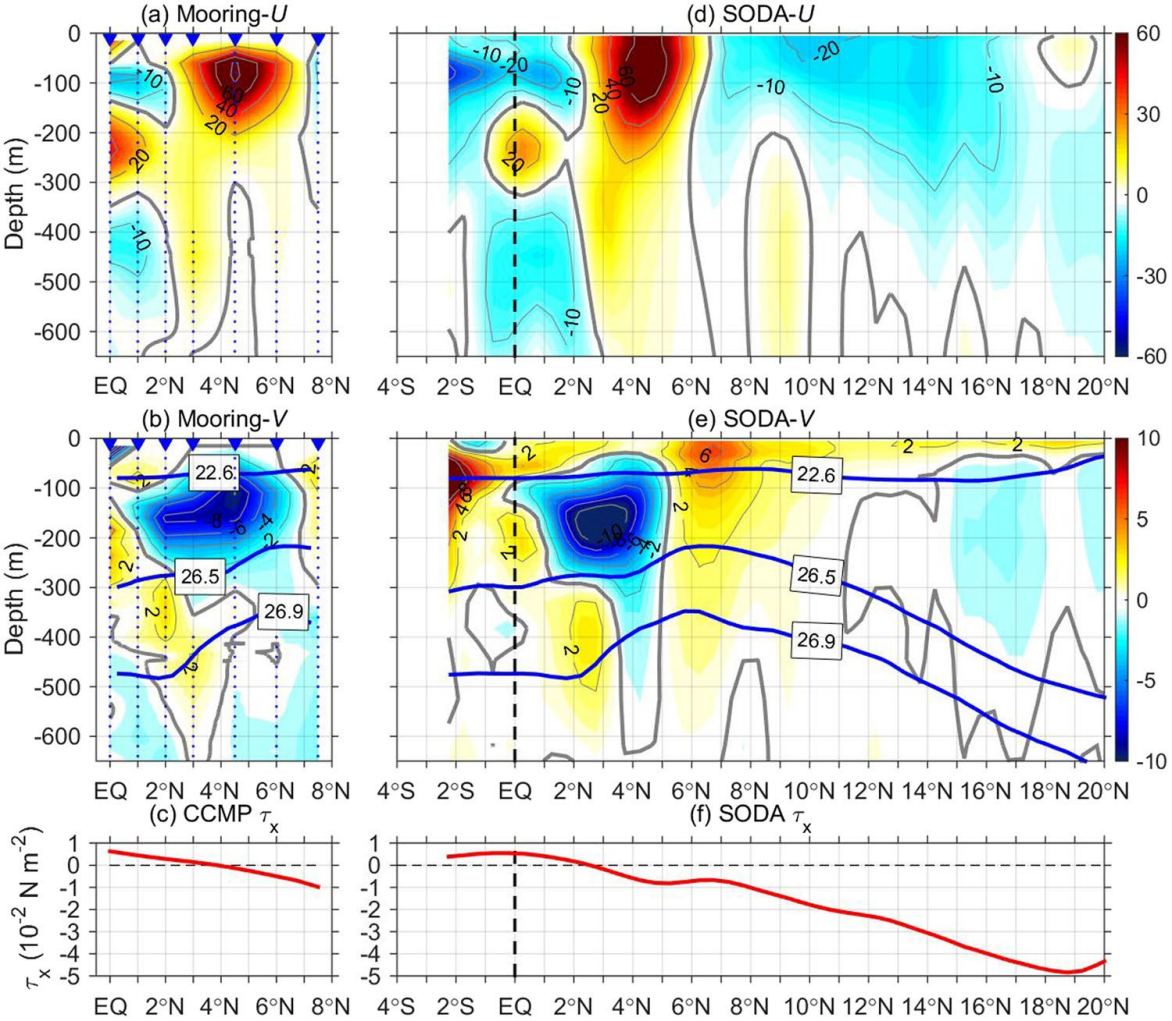
Upper-ocean currents and local zonal wind stress. (**a**) Mean zonal velocity (*U*; in cm s^−1^) and (**b**) meridional velocity (*V*; in cm s^−1^) during August 2014-October 2015, based on OSCAR surface current data and ADCP measurements of subsurface moorings along 142°E. Blue triangles denote mooring locations, and gray dots denote the coverage of ADCP measurements. (**c**) Zonal wind stress (*τ*_*x*_) averaged between 140−143°E derived from CCMP. (**d**–**f**) are the same as (**a**–**c**) but derived from SODA product, averaged between 140–143°E. Blue thick contours in (**b**) and (**e**) denote the 22.6 *σ*_*θ*_, 26.5 *σ*_*θ*_ and 26.9 *σ*_*θ*_ isopycnal surfaces, derived from SODA data, which are used to define different layers of the TMOC. The figure is plotted using MATLAB R2014b (http://www.mathworks.com/).

The mean ocean current during August 2014-October 2015 at 142°E was obtained from the interpolated mooring observational data (Fig. 1). The *U* field between 0–6°N clearly captured the following three major eastward equatorial currents (Fig. 1a): the eastward North Equatorial Countercurrent (NECC) with a velocity core >80 cm s^−1^ at 4.5°N, 80 m; the EUC between 150–350 m near the equator; and the Northern Subsurface Countercurrent (NSCC) centered at 3°N, 400 m. The westward flowing currents included the northern branch of the South Equatorial Current (SEC) above 130 m and the Equatorial Intermediate Current (EIC) below 350 m.

For the first time, the time-mean subsurface meridional current associated with the TMOC was documented by *in situ* observations in the western Pacific (Fig. 1b). The mean meridional current is generally much weaker than the zonal current. In the surface layer, a weak northward current existed above 22.6 kg m^−3^ potential density surface (*σ*_*θ*_) with a magnitude of ~2 cm s^−1^, extending from the equator to ~3°N. The surface poleward flow, which corresponds to the equatorial divergence, brings the SEC water to the NECC^20^. A strong southward flow was present in the thermocline (22.6–26.5 *σ*_*θ*_) over a wide latitude range of 1–6°N. A maximal southward current of >8 cm s^−1^ was observed at 4.5°N, 150 m. This equatorward flow mainly occurs above the EUC core, brings the cold thermocline water from the lower NECC to the equator, and feeds the EUC. Below the thermocline, a northward flow with a mean velocity of ~2 cm s^−1^ was seen to extend from the equator to ~4°N between 26.5–26.9 *σ*_*θ*_. This flow provides a pathway for the water mass exchanges between the NSCC and lower portion of the EUC. The subthermocline poleward flow is also found in the eastern Pacific TMOC by Wang^17^, who believed that it is closely associated with the equatorial dynamics and plays an essential role in the formation of the NSCC. This weak subsurface northward current exists not only in the observational period but also in long-term climatology (Figs S1 and S2 and figures not shown).

The vertically sandwiched, three-layer meridional currents indicate that the TMOC has a double-cell structure. This structure is dramatically different from the single-cell TMOC suggested in previous modeling studies^6,8,18^. Thermocline off-equatorial water joins the EUC and then bifurcates vertically, leaving the equator through both surface and subthermocline poleward flows. Compared to the upper cell, the lower cell is of relatively smaller strength, and thus has not received much attention^7,21,22^. Equatorial upwelling and downwelling seem to occur above and below the EUC velocity core (at ~250 m), respectively. Such a reversal has also been reported in the central-to-eastern Pacific basin^23,24^. The off-equatorial downwelling branch of the TMOC appears approximately within the vicinity of the NECC (3–6°N), and subsurface upwelling might exist beneath the NECC. It is interesting to note that there is a northward shoaling of the surface poleward current and a northward deepening of the subthermocline poleward current. According to the established theoretical context, the TMOC is a basin-wide overturning cell driven by equatorial easterly winds^3,25^. However, in the far western Pacific, there are mean westerly winds at the equator (Fig. 1c), which suggests that the observed TMOC at 142°E cannot be explained by local winds, and that remote forcing of the easterly winds in the central and eastern Pacific should be involved in the formation of the western Pacific TMOC.

The mean zonal and meridional currents and surface wind stress derived from SODA data (Fig. 1d–f) compare favorably with the mooring observations. Importantly, the sandwiched TMOC also shows up in SODA. This sandwiched *V* structure also exists south of the equator, albeit with a lower strength for the lower cell. SODA further suggests that the double-cell structure is a common feature of the Pacific TMOC, and is discernible in the western, central and eastern parts of the Pacific (Fig. S1). Moreover, additional equatorward convergent flow is seen in the intermediate layer below 400 m, corresponding to the depths of the EIC, which is also discernible from the mooring observations (Fig. 1b). Such vertically alternating convergence and divergence of meridional currents are reminiscent of vertical normal modes, which are dominant in the equatorial ocean response to wind forcing. However, addressing the dynamics underlying the generation of this interesting structure is beyond the scope of this study. Using current data from OSCAR and SODA, the horizontal distribution of *V* in different layers can be displayed (Fig. S2). In the surface and thermocline (22.6–26.5 *σ*_*θ*_) layers, *V* shows basin-wide equatorial divergence and convergence patterns over the Pacific basin respectively, whereas the subthermocline *V* (26.5–26.9 *σ*_*θ*_) is much noisier and exhibits large spatial variations, indicating that the interpretation of the flow structure in this layer should be with caution.

### Variations of the TMOC and Its Impact on SST

**Figure 2:**
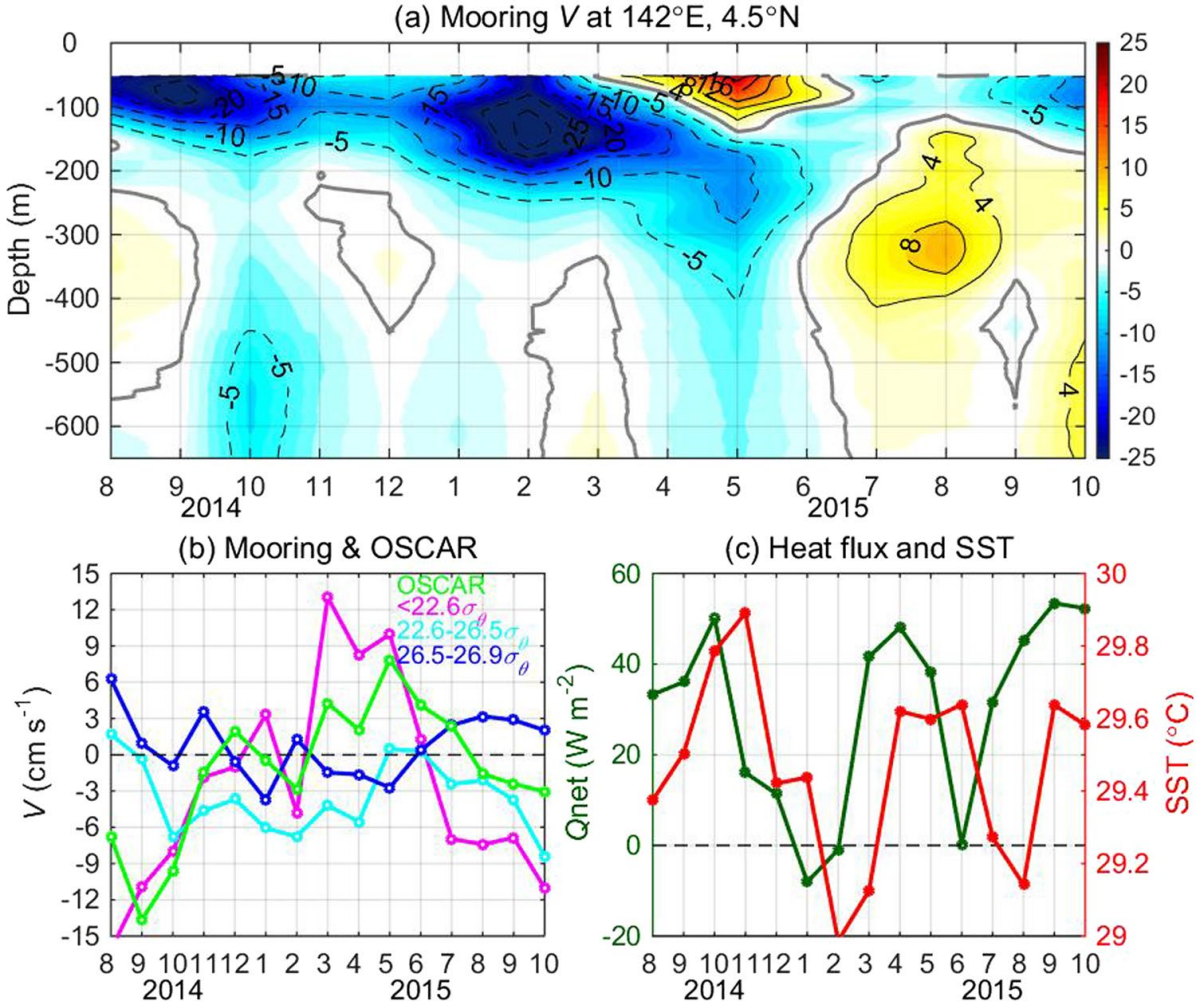
(**a**) Time-depth plot of ADCP-measured monthly *V* (cm s^−1^) derived from the mooring at 142°E, 4.5°N. (**b**) Mooring-measured monthly *V* at 142°E, 1–6°N averaged over surface layer (<22.6 *σ*_*θ*_), thermocline layer (22.6-26.5 *σ*_*θ*_), and subthermocline layer (26.5–26.9 *σ*_*θ*_). Green curve denotes OSCAR surface *V* averaged between 140–143°E. (**c**) Monthly SST and net surface heat flux *Q*_net_ averaged between 140–143°E, 2°S-2°N. The figure is plotted using MATLAB R2014b (http://www.mathworks.com/).

Prominent variability was observed at all the mooring sites. For example, the mooring at 4.5°N documented strong variations in all three layers of the TMOC (Fig. 2a). The surface northward current did not reach the upper limit of the observation range (60 m) until March 2015, showing a southward peak of 30 cm s^−1^ in September 2014 and a northward peak of >20 cm s^−1^ in May 2015. The southward thermocline current achieved a maximum of 30 cm s^−1^ in February-March 2015; in summer 2015, it was greatly weakened and partly replaced by a northward current. The subthermocline *V* showed frequent reversals during the observation period and reached a maximum strength of 4–9 cm s^−1^ in summer 2015. These results suggest that the TMOC exhibits strong and complex variations, and that it is necessary to quantify the variations separately for different layers (Fig. 2b). Averaged over 1–6°N, the OSCAR data-based surface *V* is consistent with the mooring-based surface-layer *V* (<22.6 *σ*_*θ*_). They reached a northward peak of 5–13 cm s^−1^ in March-May 2015 and a southward peak of >14 cm s^−1^ in summer 2014, yielding a peak-to-peak difference of ~27 cm s^−1^. In comparison, the variations in the thermocline and subthermocline layers are weaker, and no consistent relationship can be discerned between these two layers during the observation period. However, the surface *V* and thermocline current between 22.6–26.5 *σ*_*θ*_ show in-phase variations during the following two periods: in summer 2014, the TMOC was reversed with a southward surface flow and a northward thermocline flow; and in the spring of 2015, the TMOC was strengthened in its upper cell with a strong surface northward flow and a southward thermocline flow. The lack of consistent relationships between different layers might be because during the observation period, signals of seasonal and interannual variability were intermixed, and the characteristics of these two types of variability may differ.

The potential impact of the TMOC on SST is of particular interest. During the observation period, equatorial SST in the western Pacific showed pronounced variations with an amplitude of 0.5 °C (red line in Fig. 2c). Most of the variation in SST can be explained by net surface heat flux *Q*_net_ (green line in Fig. 2c), with SST following *Q*_net_ by 1–2 months. Nevertheless, effects of the TMOC can be seen during some seasons. For instance, *Q*_net_ reached similar magnitudes in October 2014 and April 2015, whereas the SST peak in November 2014 was higher than that in spring 2015 by ~0.3 °C. This difference can likely be ascribed to the equatorial downwelling (upwelling) of the weakened (strengthened) TMOC during summer 2014 (spring 2015).

**Figure 3:**
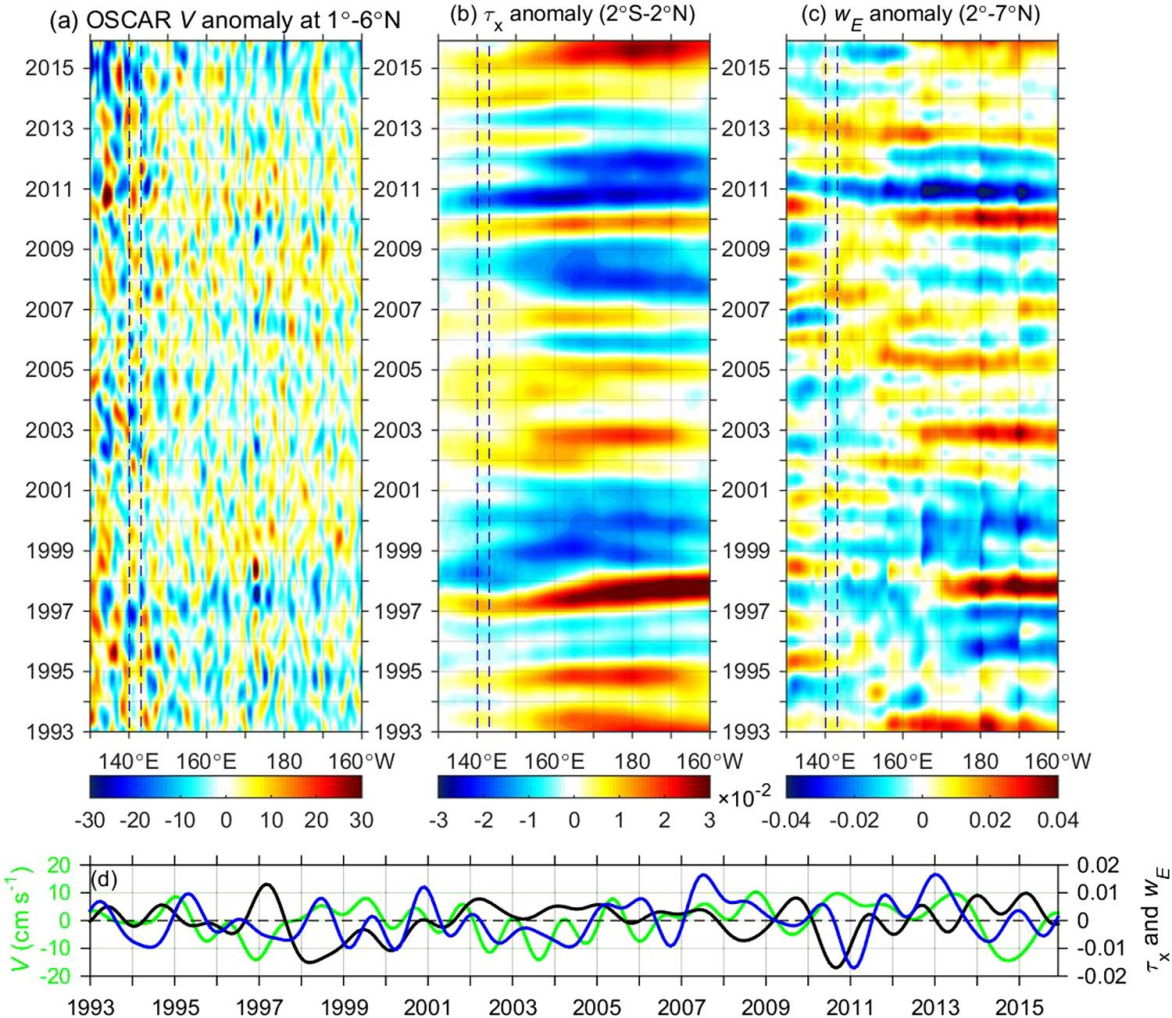
Time-longitude plots of (**a**) OSCAR *V* averaged between 1–6°N (cm s^−1^), (**b**) equatorial *τ*_*x*_ (N m^−2^; 2°S-2°N average), and (**c**) off-equatorial Ekman pumping velocity *w*_E_ (10^−4^ m s^−1^; 2–7°N average). (**d**) Monthly time series of (**a**–**c**) averaged between 140–143°E, with green for OSCAR *V*, black for *τ*_*x*_, and blue for *w*_E_, respectively. All the variables are shown as 13-month low-pass filtered anomalies. *τ*_*x*_ and *w*_E_ are based on CCMP wind data. The figure is plotted using MATLAB R2014b (http://www.mathworks.com/).

Due to the failure of El Niño development in 2014 and the 2015–2016 “super El Niño” event, the observation period was generally under the El Niño condition. We plotted time-longitude diagrams of 13-month low-pass filtered OSCAR *V*, zonal wind stress (*τ*_*x*_) and Ekman pumping velocity to explore the impact of ENSO on interannual TMOC variations (Fig. 3). For the 1993–1995, 1997, 2002–2006, and 2014–2015 periods, strong negative *V* anomalies between 1–6°N, 140–143°E stood out, which indicated a weakened surface branch of the TMOC (Fig. 3a). These periods are all characterized by strong El Niño or El Niño-like conditions. The basin-scale equatorial westerly anomalies (2°S-2°N) during these periods (Fig. 3b) caused equatorial surface convergence and downwelling and thereby weakened the TMOC throughout the basin. This explains the overall weak TMOC strength recorded in our mooring observations (Fig. 2b). In contrast, for the 1998–2000, 2008, and 2010–2011 periods, easterly anomalies of the La Niña condition forced surface divergence and upwelling at the equator and strengthened the TMOC.

The correlation coefficient between surface *V* and equatorial *τ*_*x*_ is −0.47 (significant at the 95% confidence level), indicating that equatorial zonal winds exert a strong forcing effect on the equatorial upwelling branch of the TMOC. On the other hand, off-equatorial winds primarily affect the downwelling branch of the TMOC through Ekman pumping (Fig. 3c). A positive (negative) *w*_E_ anomaly drives upwelling (downwelling) off-equatorial Rossby waves and weakens (strengthens) the TMOC; therefore, a negative correlation between off-equatorial *w*_E_ and surface TMOC is expected. However, the correlation coefficient between surface *V* and 2−7°N averaged *w*_E_ is positive (0.29; Fig. 3d), suggesting that off-equatorial winds generally act to attenuate TMOC variations. In addition, a westward propagation tendency is not evident in the surface *V*, indicating no Rossby wave signatures. The composite surface *V* anomaly for the El Niño condition suggests a weakened TMOC, manifesting as surface convergence toward the equator over the whole basin (Fig. S3). The situation is opposite for the La Niña composite. To summarize, anomalous equatorial zonal winds associated with ENSO play a dominant role in modulating the interannual variability of the Pacific TMOC.

**Figure 4:**
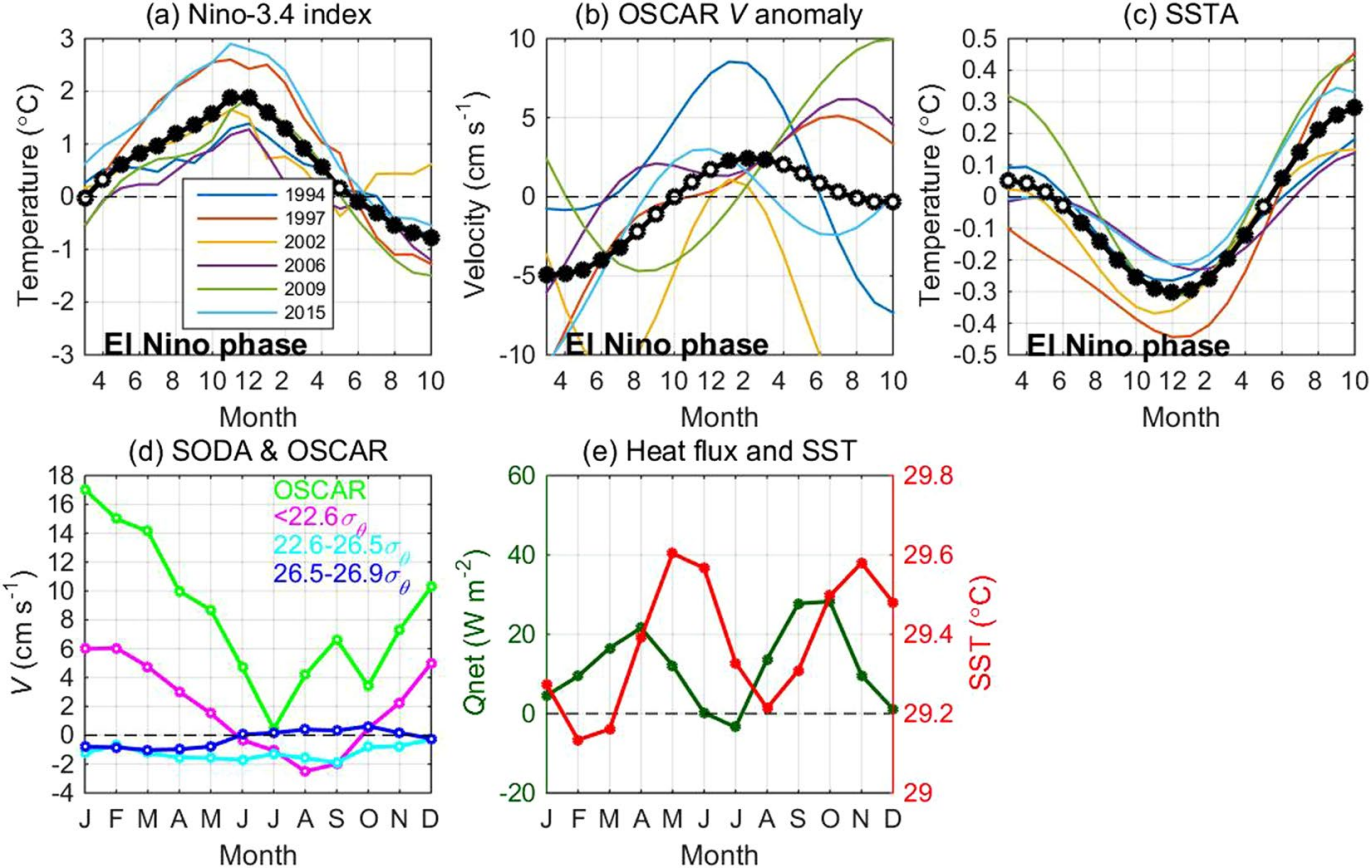
Evolutions of 13-month low-pass filtered (**a**) Niño-3.4 index, (**b**) OSCAR *V* anomaly (1–6°N), and (**c**) equatorial SST anomaly (2°S-2°N) for the El Niño events during 1993–2016. The black curve denotes the composite, with black and white dotes denoting composite values above and below the 90% confidence level based on a students’ *t*-test, respectively. (**d**) Monthly climatology of OSCAR *V* and SODA *V* in different layers and (**e**) equatorial *Q*_net_ and SST from SODA (2°S-2°N). All the variables from (**b**–**e**) are averaged between 140–143°E. The figure is plotted using MATLAB R2014b (http://www.mathworks.com/).

To examine the impact of TMOC on interannual equatorial SST, we checked the evolution of six El Niño events (Fig. 4a). From the initial development to the peak stage of an El Niño event, the composite surface *V* anomalies of OSCAR and SODA change from negative to positive (Figs 4b and S4). In response, the equatorial SST of the western Pacific shows a weak warming in the developing stage and a strong cooling at the peak stage of the El Niño (Fig. 4c). The enhanced TMOC may cool the western Pacific SST by entraining cold thermocline water to the equatorial surface mixed layer and contribute to the formation of the seasaw SST pattern of ENSO. In addition, the weakened TMOC in spring-summer, in response to westerly wind bursts, also may contribute to the initial SST warming over the western-to-central Pacific.

Because the mooring observations are greatly influenced by the El Niños, we are unable to extract robust seasonal TMOC variations. We therefore examined the monthly climatology of OSCAR surface *V* and SODA surface *V* to gain insights into TMOC seasonality in the western Pacific (Fig. 4d). The surface component is stronger in winter than in summer by >9 cm s^−1^, showing strong seasonal variation. Seasonal variations of the thermocline and subthermocline components are much weaker, with amplitudes of only ~2 cm s^−1^. The enhanced TMOC contributes to the formation of the SST minimum in February-March (Fig. 4e), although the semiannual SST cycles are mainly forced by *Q*_net_. *Q*_net_ reaches a minimum in July; however, it does not lead to the SST minimum over a year (weaker magnitude than in February-March), which is due to the weakened TMOC. These findings indicate that upwelling of the TMOC plays a role secondary to surface heat flux forcing in modulating the western Pacific SST change, which is consistent with our mooring observations (Fig. 2).

## Summary and Discussion

In the present study, we investigate the structure and variability of the TMOC in the northwestern Pacific by analyzing ADCP data from seven subsurface moorings at 142°E along with satellite observation, ocean reanalysis data, and atmospheric surface fields. Mooring observations during August 2014-October 2015 revealed a sandwiched, three-layer structure of the TMOC between the equator and 6°N, with northward, southward, and northward flows in the surface, thermocline, and subthermocline layers, respectively. The local surface winds are westerly in climatology and thus cannot explain the mean TMOC. The TMOC exhibits pronounced variability, and interannual variations are mainly induced by equatorial zonal wind stress changes associated with ENSO. Westerly anomalies during the El Niño condition suppress equatorial upwelling across the Pacific basin, leading to an overall weak TMOC during the observation period. The satellite and reanalysis data suggest a well-defined TMOC seasonal cycle that is stronger in winter and weaker in summer. The SST variability of the western equatorial Pacific is largely determined by surface heat flux forcing, particularly for its seasonality, and is to a lesser extent affected by the upwelling of TMOC. The weakened (strengthened) TMOC during the developing (peak) phase of an El Niño event causes SST warming (cooling) in the western Pacific and thereby plays a vital role in ENSO dynamics.

The structure of the TMOC is complicated and is rarely observed in the western Pacific, where strong winds blow over complex topography. Compared with the central-to-eastern Pacific, both the mean TMOC and its anomaly are noisier in the far western Pacific, and they exhibit meridional strip structures with a width of several degrees of longitude (Figs S2 and S3). These characteristics are potentially due to the effects of topography and the meandering of the NECC and EUC. The TMOC structure is therefore sensitive to the choice of observational position. In this study, we mainly addressed the strength variability of TMOC, while the thickness variations of the three layers are not examined. In fact, the vertical ranges of three layers show large temporal variations. For instance, the vertical ranges of thermocline northward current and subthermocline southward current fluctuated between 60–500 m in mooring observation (Fig. S5), indicating dramatic and complex changes in the TMOC structure, which requires careful quantification.

The TMOC was stronger in spring 2015 and weaker in fall 2014 and fall 2015, which appears to be inconsistent with the climatological seasonal cycle based on OSCAR and SODA data. In addition to the impact of ENSO, ocean internal variability, such as mesoscale eddies and the NECC meandering, may have contaminated these variations. For example, the strengthened TMOC at 142°E in spring 2015 was likely influenced by an anticyclonic eddy to the east that was associated with the NECC meandering (Fig. S6). A much longer observation period is required to sufficiently average out the ocean internal variability signals and obtain a robust seasonal cycle. Albeit with data assimilation, SODA still shows many discrepancies in TMOC structure and variability from the mooring observations, particularly for the thermocline and subthermocline branches (Figs 4d and S4). These discrepancies indicate that ocean models have difficulties in accurately representing the dynamical processes involved in the meridional overturning flow in the western Pacific. The mooring observations conducted in this region improve our understanding of the three-dimensional ocean circulation and can be used to refine ocean models for simulation and prediction.

## Methods

Seven subsurface mooring systems were deployed in August 2014 along 142°E at latitudes of 0°N, 1°N, 2°N, 3°N, 4.5°N, 6°N and 7.5°N, and were recovered in October 2015. Each mooring was equipped with one upward-looking and one downward-looking 75-kHz ADCP within its main float at a design depth of 400 m. Zonal and meridional velocities (*U* and *V*) were interpolated to 10-m intervals between 60 m and 650 m and further averaged into daily mean data. Due to the failure of two upward-looking ADCPs, no data were available for the upper 400 m at 3°N and 6°N. The availability of the ADCP measurements is displayed in Fig. 1a,b. We filled the data gaps using a method similar to that of Shu *et al*.^26^. The missing data between 60–220 m were filled through linear interpolation with reference to Ocean Surface Current Analyses - Real Time (OSCAR) current data at 30 m and current measurement data from adjacent moorings, and those between 220–400 m were obtained through linear interpolation. The monthly mean data were then used to investigate the climatologic TMOC structure and seasonal-to-interannual variability.

In addition to mooring observational data, the analysis was assisted using other oceanic and atmospheric data sets, including the ^1^/_3_° 30-m ocean current data from OSCAR during 1993–2016^27,28^, 1° SST data from the HadISST v1.1^29^, 0.75° net surface heat flux (*Q*_net_) data from the European Centre for Medium-Range Weather Forecasts Interim reanalysis (ERA-Interim)^30^, 0.25° 10-m wind data from monthly Cross-Calibrated Multiplatform (CCMP) version 2.0^31^, Niño-3.4 index from the Climate Prediction Center of the National Oceanic and Atmospheric Administration (NOAA), and 0.25° data from the Simple Ocean Data Assimilation reanalysis (SODA) 3.3.1 product during 1980–2015^32^. We calculated Ekman pumping velocity (*w*_E_) with CCMP wind stress (***τ***) using *w*_E_ = *ρ*^−1^curl(***τ***/*f*), where *ρ* = 1021 kg m^−3^ is the mean density, and *f* is the Coriolis parameter.

### Data sources

The subsurface mooring data used here are available in this published article. OSCAR, CCMP and Niño-3.4 index are obtained from http://www.oscar.noaa.gov, http://podaac.jpl.nasa.gov/, and http://www.cpc.ncep.noaa.gov, respectively. HadISST and SODA products can be downloaded from the Asia–Pacific Data-Research Center at the University of Hawai’i (http://apdrc.soest.hawaii.edu/data/data.php). ERA-Interim data are obtained from the https://www.ecmwf.int/en/forecasts/datasets/reanalysis-datasets/era-interim.

## Electronic supplementary material

Supplementary file

Mooring u component

Mooring v component
