# Supplementary material for: Tropical Meridional Overturning Circulation Observed by Subsurface Moorings in the Western Pacific
Source: Sci Rep. 2018 May 16;8:7632. doi: 10.1038/s41598-018-26047-7 (PMC5955992; doi:10.1038/s41598-018-26047-7)
Supplement: Supplementary file 1 — Supplementary file [file 41598_2018_26047_MOESM1_ESM.pdf]

Supporting Information for

Tropical Meridional Overturning Circulation Observed by Subsurface Moorings in the Western Pacific

Lina Song[1,2], Yuanlong Li[1,3], Jianing Wang[1,3], Fan Wang[1,3], Shijian Hu[1,3], Chuanyu Liu[1,3], Xinyuan Diao[1], Cong Guan[1,2]

[1]Key Laboratory of Ocean Circulation and Waves, Institute of Oceanology, Chinese Academy of Sciences, Qingdao, China.

[2]Institute of Oceanographic Instrumentation, Shandong Academy of Sciences, Qingdao, China.

[3]Function Laboratory for Ocean Dynamics and Climate, Qingdao National Laboratory for Marine Science and Technology, Qingdao, China.

Contents of this file

Figure S1
Figure S2
Figure S3
Figure S4
Figure S5
Figure S6

Introduction

Four figures are uploaded as supporting information. Figure S1 shows depth-latitude plots of annual-mean climatologic current derived from SODA during 1980-2015. Figure S2 shows horizontal distribution of climatologic *V* in the Pacific Ocean. Figure S3 composites OSCAR surface *V* anomaly for El Niño and La Niña conditions, respectively. Figure S4 composites depth-time evolution of SODA *V* anomaly for the El Niño events. Figure S5 shows vertical ranges of the northward current and southward current. Figure S6 shows seasonal-mean OSCAR surface current anomaly and its meridional component from fall 2014 to fall 2015.

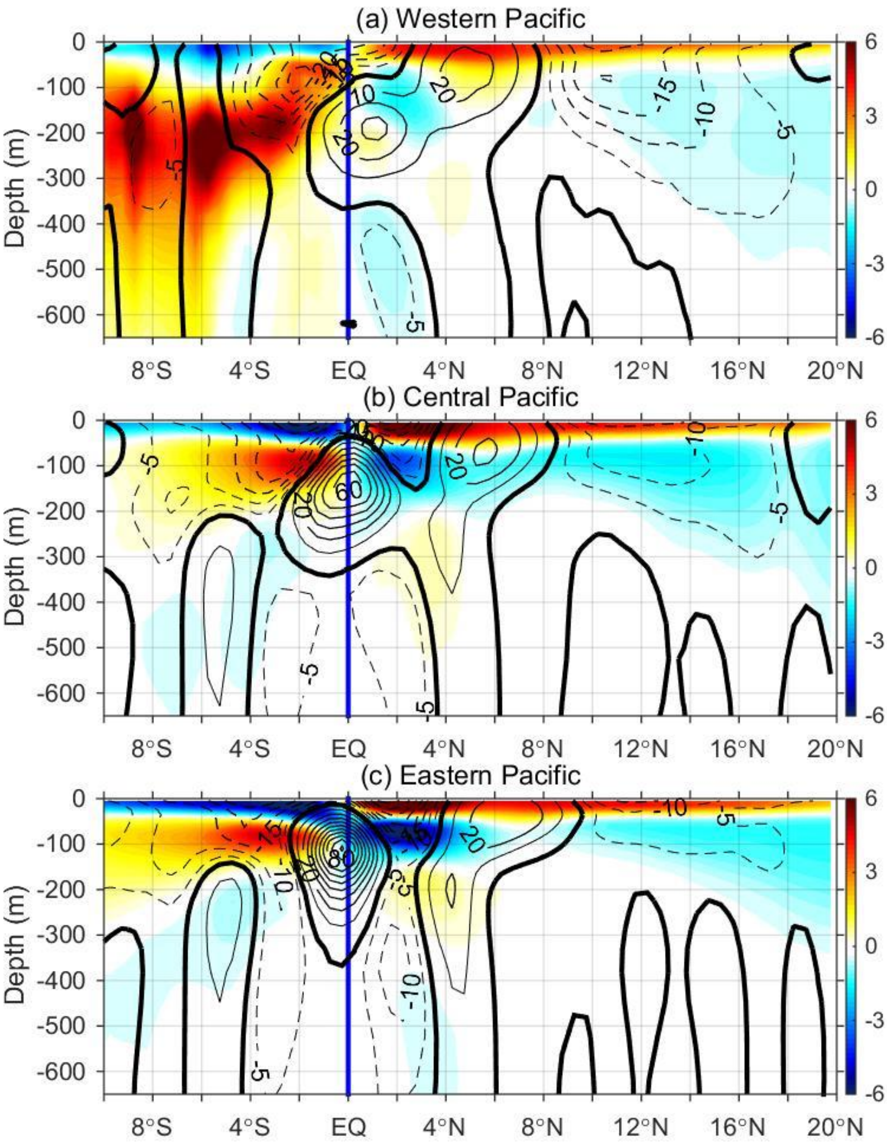

Figure S1. Depth-latitude plots of annual-mean climatologic current derived from SODA during 1980-2015 in (a) the western Pacific (140°-160°E), (b) central Pacific (180°-160°W) and (c) eastern Pacific (150°-130°W). Color shading is meridional velocity (*V*; cm s^{-1}), and black contours are zonal velocity (*U*; cm s^{-1}; contour interval is 10 cm s^{-1} for positive and 5 cm s^{-1} for negative). The figure is plotted using MATLAB R2014b (http://www.mathworks.com/).

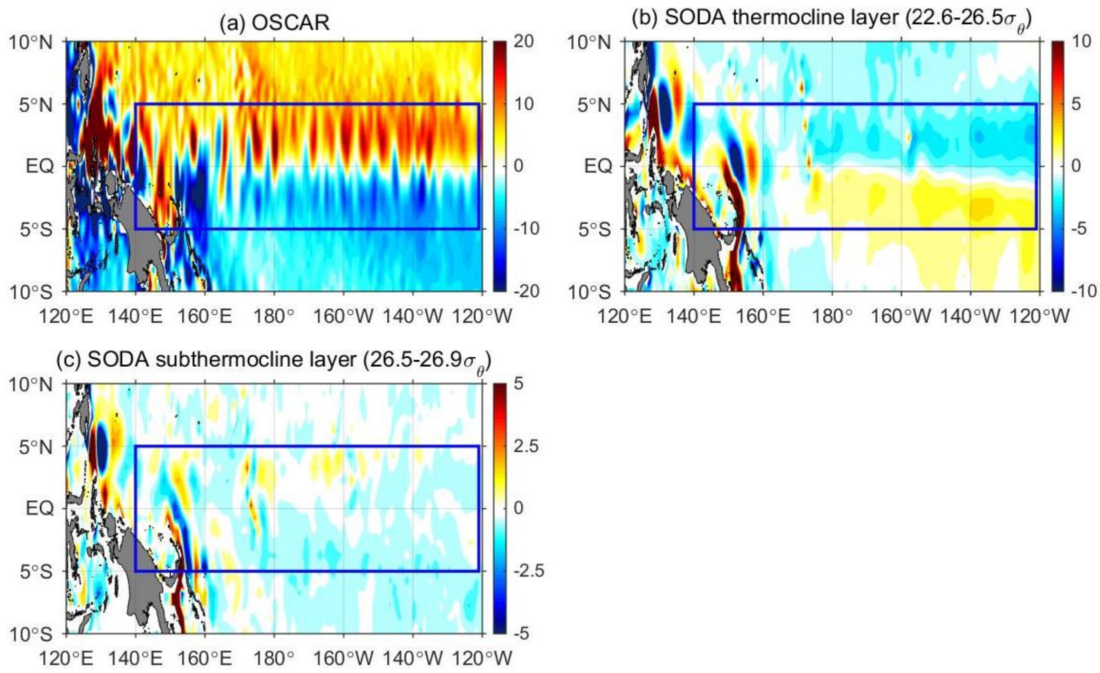

Figure S2. Horizontal distribution of climatologic *V* (cm s^{-1}) in the Pacific Ocean. (a) shows OSCAR surface *V* for 1993-2016, while (b)-(c) show the average *V* of (b) thermocline layer (22.6-26.5σ_θ), and (c) subthermocline layer (26.5-26.9σ_θ) derived from SODA product during 1980-2015. The figure is plotted using MATLAB R2014b (http://www.mathworks.com/). The maps in this figure are generated by MATLAB R2014b with Global Relief Model data of ETOPO1[1] (https://www.ngdc.noaa.gov/mgg/global/global.html).

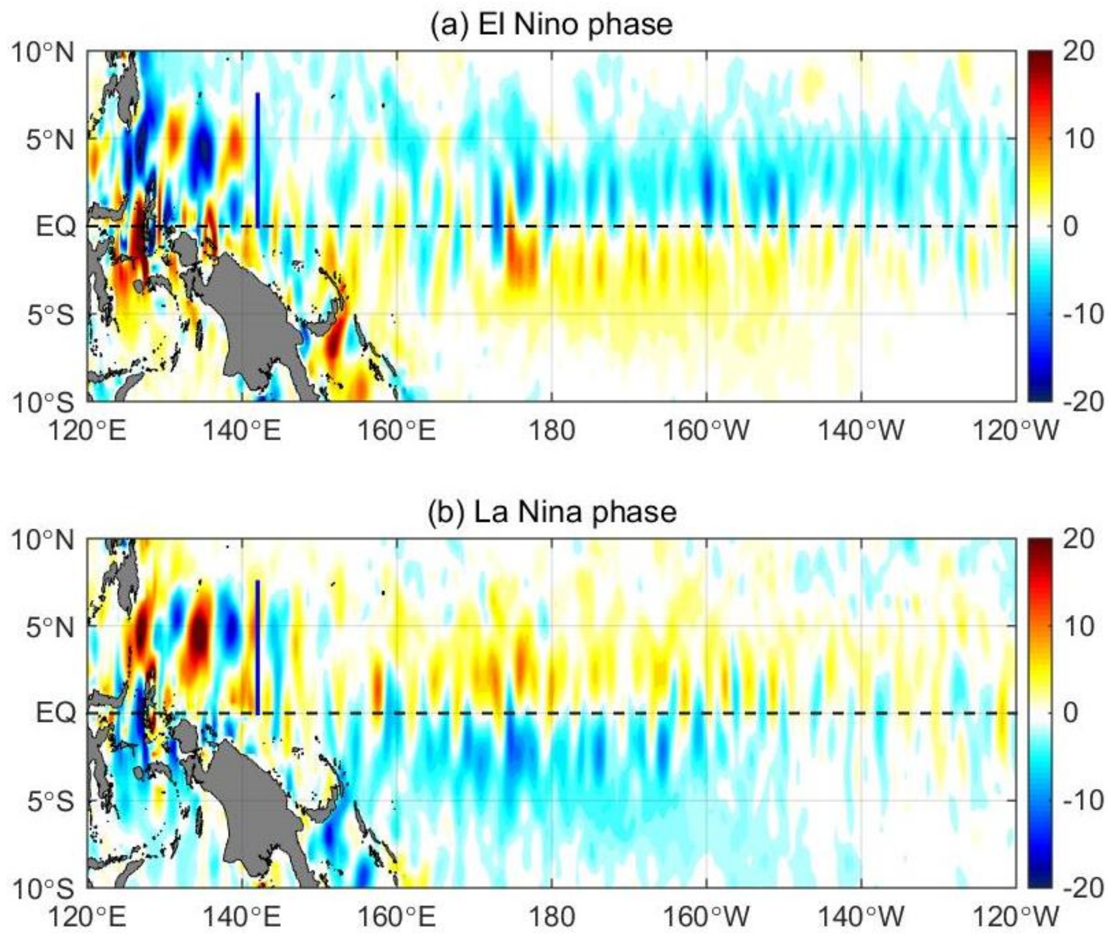

Figure S3. (a) Composite OSCAR surface *V* anomaly (cm s^{-1}) for El Niño condition when the Niño-3.4 index exceeds its +1 standard deviation during 1993-2016. *V* anomaly has been 13-month low-pass filtered. (b) The same as (a) but for La Niña condition when the Niño-3.4 index is lower than -1 standard deviation. The figure is plotted using MATLAB R2014b (http://www.mathworks.com/). The maps in this figure are generated by MATLAB R2014b with Global Relief Model data of ETOPO1[1] (https://www.ngdc.noaa.gov/mgg/global/global.html).

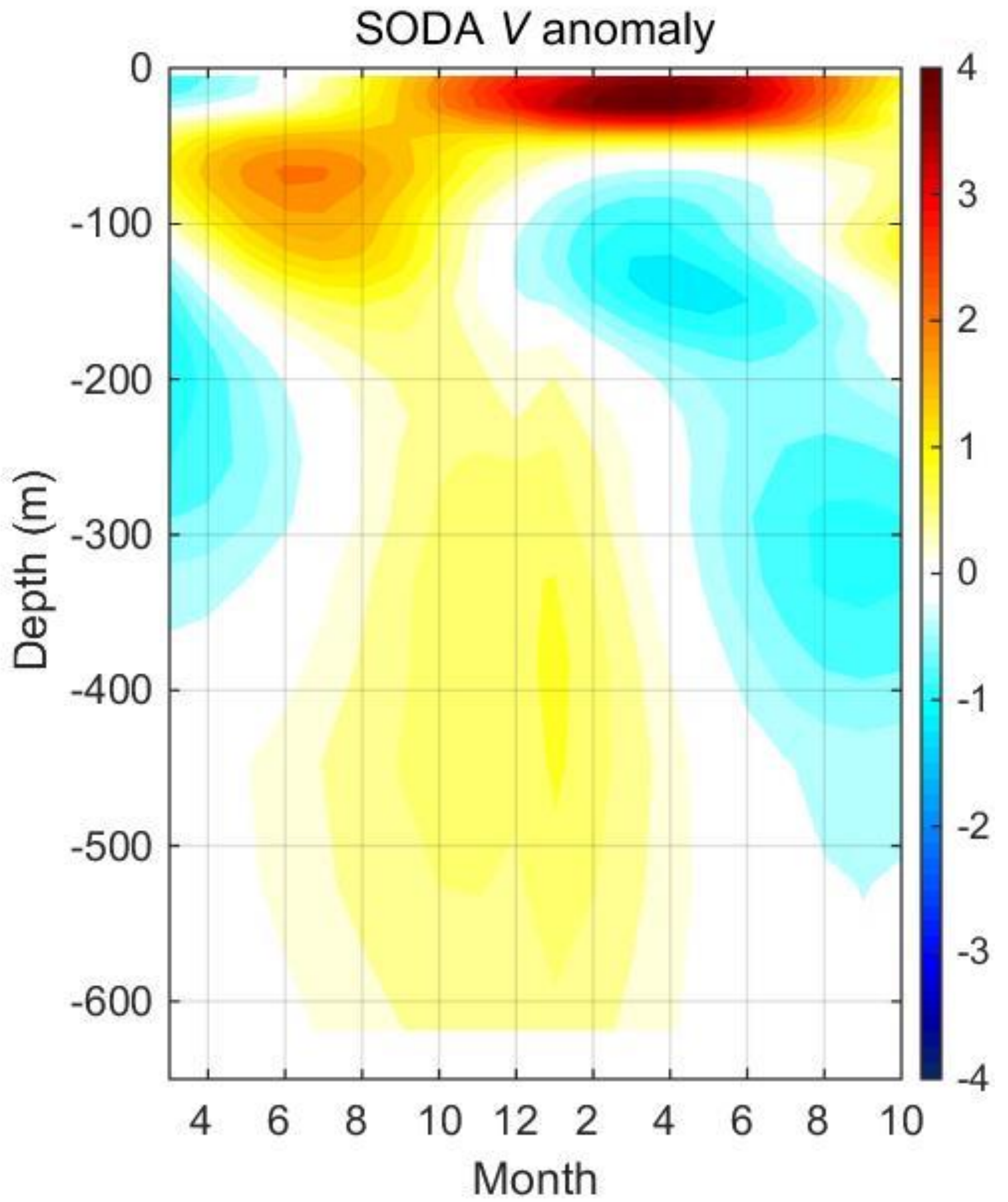

Figure S4. Depth-time evolution of 13-month low-pass filtered SODA *V* anomaly (140°-143°E, 1°-6°N; in cm s^{-1}) for the El Niño events during 1993-2015. The figure is plotted using MATLAB R2014b (http://www.mathworks.com/).

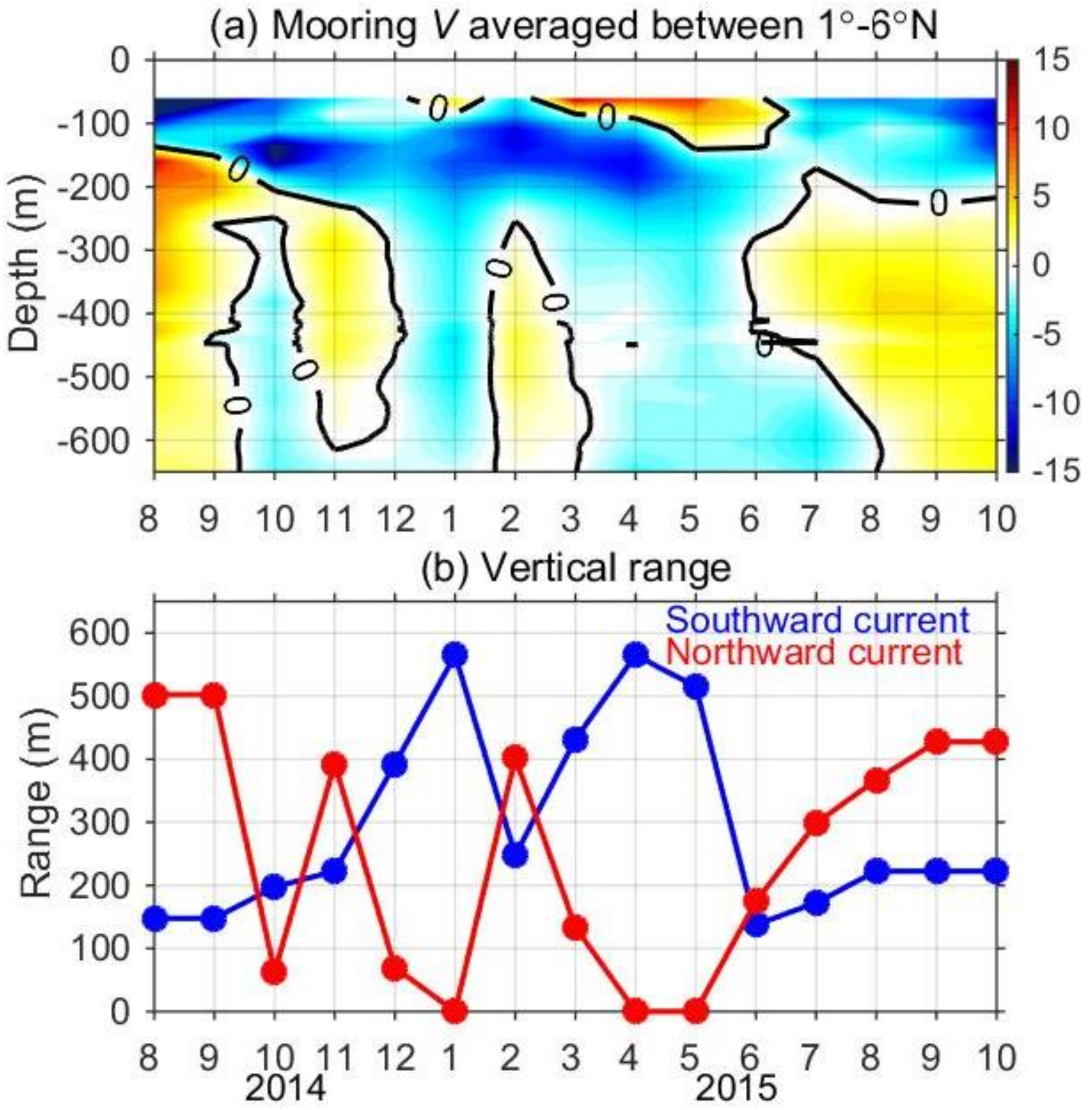

Figure S5. (a) Time-depth plot of ADCP-measured monthly *V* (cm s^{-1}) averaged between 1°-6°N. (b) Monthly vertical ranges (m) of the northward current below 150 m (red line) and southward current (blue line) from (a). The figure is plotted using MATLAB R2014b (http://www.mathworks.com/).

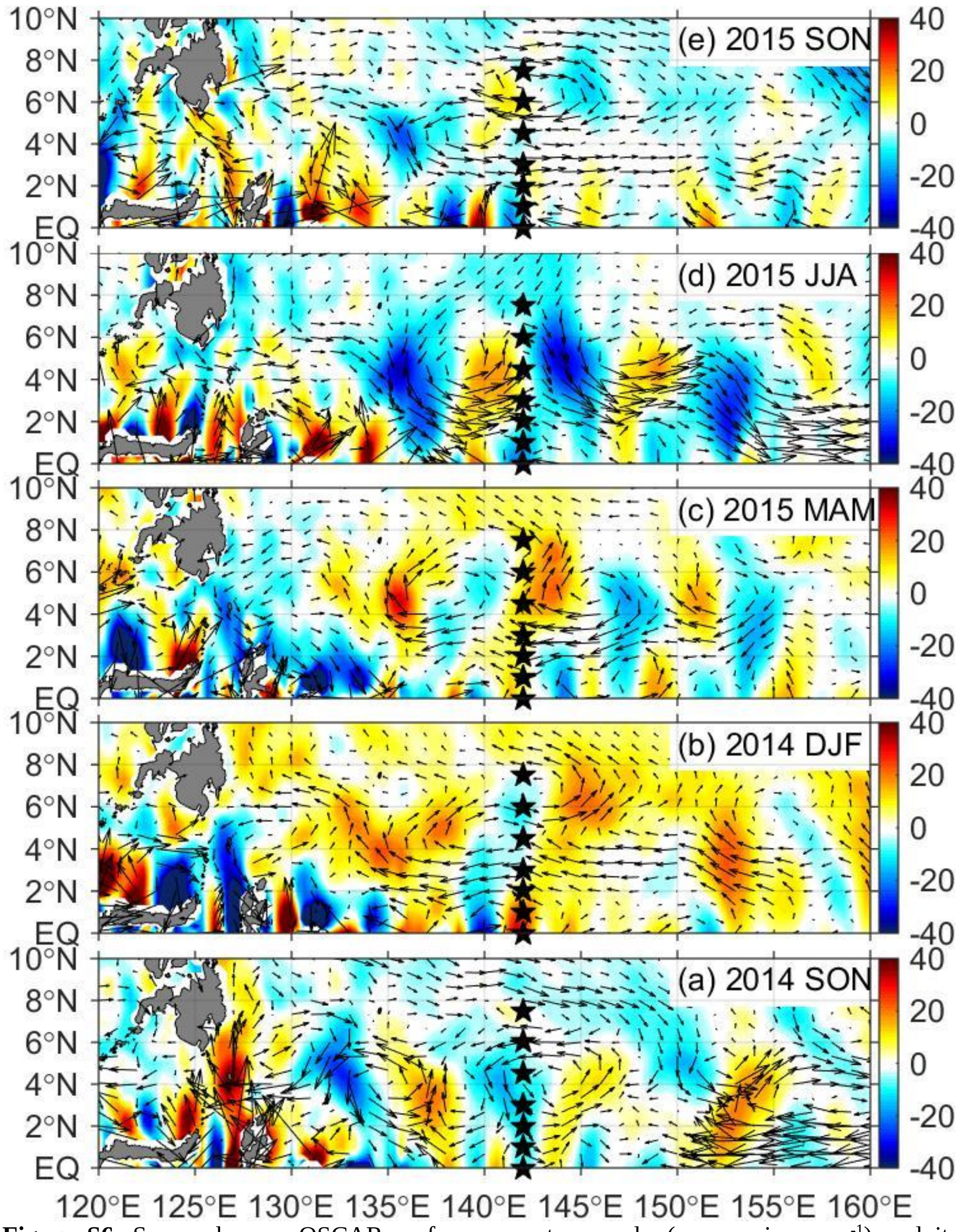

Figure S6. Seasonal-mean OSCAR surface current anomaly (arrows; in cm s^{-1}) and its meridional component (color shading; in cm s^{-1}) from fall 2014 to fall 2015 (from bottom to top). Mooring locations are marked as black stars at 142°E (0°N, 1°N, 2°N, 3°N, 4.5°N, 6°N, and 7.5°N). The figure is plotted using MATLAB R2014b (http://www.mathworks.com/). The maps in this figure are generated by MATLAB R2014b with Global Relief Model data of ETOPO1[1] (https://www.ngdc.noaa.gov/mgg/global/global.html).

References

1. Amante, C. & Eakins, B. W. ETOPO1 1 Arc-Minute Global Relief Model: Procedures, Data Sources and Analysis. NOAA Technical Memorandum NESDIS NGDC-24. National Geophysical Data Center, NOAA, doi:10.7289/V5C8276M (2009).
